# Rhinoplastic Operation

**Published:** 1851-07

**Authors:** 


### ARTICLE XXIV.

### *Rhinoplastic Operation.*

Mr. Fergusson performed this operation at King's College Hospital, in February, on a man of about forty, who had been under his care several times before. His nose had suffered very much, and nothing but a shriveled, flattened and compressed little mass was seen in the site of the original organ, the left ala being most depressed. This destruction had originally been caused by a blow, from which the ossa nasi and cartilages had suffered severe injury. The parts had healed very slowly, and Mr. Fergusson, a twelvemonth ago, had made incisions near the eyebrows for the removal of necrosed bones. At that time, the man was promised that a new nose would be made for him. He had gone into the country, where he recruited his health ; and now had come to have the operation performed.

Mr. Fergusson gave, in this instance, the preference to the Indian method ; and the patient having been placed under the influence of chloroform, a triangular piece of leather, cut into the shape of the new organ, and made to suit the irregularities of the stump, was spread flat upon the forehead, with its base uppermost ; deep incisions, with subsequent parings, were then made along the margins of the deformed nose, following the line where the sides of the flap, to be presently cut from the forehead, were to be implanted. When the paring had been carefully and regularly done on both sides, Mr. Fergusson cut out the skin and cellular tissue of the forehead, down to the periosteum, according to the shape above mentioned, and this being carefully dissected from above, downwards to the root of the nose, where the dissection was carried deep, to render the vascular connexion more extensive, the flap was twisted on itself, and its edges were brought in contact with the grooves previously made. An opening was here seen into the frontal sinus from the old exfoliation. The hemorrhage was rather considerable, and somewhat increased the already great amount of trouble which this operation entails upon the surgeon ; the sutures were, however, very neatly applied, three on each side ; they kept the transplanted structure very steadily in situ, and the angles of the raw surface on the forehead, were likewise approximated by sutures. The cavity of the new nose, which was partly supported by the shriveled remains of the old, was borne up by a small quantity of lint ; the same was likewise put on the raw surface of the forehead ; the parts were carefully and warmly wrapped up, and the patient removed.

Mr. Fergusson, in addressing the assembled pupils, remarked, that out of the various materials with which noses might be replaced, the patient had chosen his own skin, and that he had thought it right to comply with his request; the more so, as he was a favorable subject for the operation, since the destruction of the organ had originally been caused, not by an idiopathic disease, but by a blow. The operation, as far as it had now gone, was only done in the rough; there was a great deal to shape out yet, until the new organ should be brought to a proper form. Mr. Fergusson observed, that this would be an extremely painful proceeding, were it not for the great boon conferred by chloroform, and that Mr. Liston had performed the same operation soon after the anesthetic use of ether had been introduced.

The patient, seven days after the operation, was attacked by erysipelas; he was delirious at intervals, particularly at night; the eyes and cheeks swelled considerably, and the new nose turned of a dull, reddish color. Still, the wound of the forehead went on contracting and granulating nicely. The delirium became soon so violent, that the patient was, at one time, hardly prevented from tearing off the new nose; it was, therefore, found necessary to use the straight jacket, and to give him a male watcher. He, however, became gradually quieter, and about eighteen days after the operation, he was more calm, dosing almost incessantly; his face assumed a more natural and sane expression, but when spoken to, he gave no answer, and appeared to be deaf. The treatment of the erysipelas and violent symptoms consisted principally in stimulants, as chloric ether and ammonia, opium, wine, &c. The nose, however, and the forehead, were looking very well; the latter was fast cicatrising, and the former seemed to have adhered firmly, especially at the root. The opening into the frontal sinus above mentioned remains patent, and seems not disposed to fill up. Twenty-two days after the operation, the patient was removed into the physicians' ward, where, after having given a great deal of trouble with delirium tremens, he is now progressing very favorably.

We had, a few days ago, an opportunity of seeing this patient, and were highly gratified at observing the firm manner in which the transplanted flap had grown to its new situation.

The nose, though in an unfinished state, looks remarkably well, the man having, of course, quite a different physiognomy. If it were not for the falling in of the incisive portion of the upper jaw, the patient would certainly not have to complain of his looks.

It would thus appear that the rhinoplastic operation has, in this case, endangered the patient's life ; and though erysipelas had a great share in these hazardous results, it cannot be denied that this apparently slight operation is not exempt from danger ; neither are the statistics encouraging. Out of two patients, Blandin almost lost one ; Lisfranc had one death ; and Dieffenbach has recorded two fatal cases out of six. The Italian method, where the flap is taken from the arm, *prima facie* seem less liable to the complications mentioned above ; but it is so inconvenient that it has been very justly abandoned. The French method, introduced by Larrey, in which the cheeks furnish the materials, has advantages in some cases, but is likewise liable to accidents. (We had recently an opportunity of witnessing the latter method applied at St. Bartholomew's Hospital, by Mr. Lloyd ; we shall lay the case before our readers when the patient's progress has been a sufficient time under observation.) We look forward with great interest to the further operations Mr. Fergusson may judge proper to perform upon this patient—viz. whether he will form a septum from the upper lip, as proposed by Liston, or remedy the falling in of the apex by the method he has himself followed, with success, in a former case—viz. by bringing the cheeks more in the mesial line, and giving, as it were, a new foundation to the nose ; though, perhaps, a newly-made organ will not be so favorable to this mode of operation as to the plan proposed by Liston.

